# Resilience of *Chlorella vulgaris* to Simulated Atmospheric Gas Compositions of Mars, Jupiter, and Titan

**DOI:** 10.3390/life15010117

**Published:** 2025-01-17

**Authors:** Ariela Likai, Aikaterini Papazi, Kiriakos Kotzabasis

**Affiliations:** Department of Biology, University of Crete, Voutes University Campus, GR-70013 Heraklion, Crete, Greece

**Keywords:** *Chlorella vulgaris*, photosynthesis, extraterrestrial environments, Martian atmosphere, Jovian atmosphere, Titan atmosphere, metabolic plasticity

## Abstract

This study investigates the resilience of the unicellular green microalga *Chlorella vulgaris* to extreme atmospheric conditions simulating those of Mars, Jupiter, and Titan. Using Earth as a control, experiments were conducted under autotrophic and mixotrophic conditions to evaluate the organism’s photosynthetic efficiency, oxygen production, and biomass growth over 2, 5, and 12 days. Photosynthetic performance was analyzed through chlorophyll a fluorescence induction (JIP-test), metabolic activity via gas chromatography, and biomass accumulation measurements. Despite the extreme atmospheric compositions—ranging from the CO_2_-rich, low-pressure Martian atmosphere to the anoxic atmospheres of Jupiter and Titan—*C. vulgaris* demonstrated resilience and a functional photosynthetic apparatus, maintaining growth and oxygen production. Notably, the Martian atmosphere enhanced photosynthetic performance, with fluorescence curves and Fv/Fm ratios surpassing Earth-like conditions, likely due to elevated CO_2_ and low pressure. Under mixotrophic conditions, the addition of glucose further enhanced metabolic activity and biomass growth across all atmospheres. These findings highlight the potential of *C. vulgaris* for bioregenerative life support systems, enabling oxygen production, CO_2_ sequestration, and resource cultivation in extraterrestrial habitats. The study showcases the organism’s adaptability to extreme environments, with implications for astrobiology, space exploration, and sustainable extraterrestrial ecosystems. These findings expand habitability criteria and explore extremophiles’ potential to support life beyond Earth.

## 1. Introduction

Earth’s atmosphere is a gaseous layer surrounding the planet, held in place by gravity, and extends to the Kármán line at approximately 100 km above sea level, where space is conventionally considered to begin. Atmospheric drag on spacecraft becomes noticeable below altitudes of about 150 km. The boundary between the atmosphere and space is not clearly defined; it gradually thins out and dissipates into space. Earth’s atmosphere protects life by absorbing ultraviolet solar radiation, retaining surface heat through the greenhouse effect, and moderating temperature fluctuations between day and night. The composition of dry air is primarily 78.08% nitrogen (N_2_), 20.95% oxygen (O_2_), 0.93% argon, 0.04% carbon dioxide (CO_2_), and traces of other gases [1]. When comparing the atmospheric compositions of some planets in our solar system (excluding their densities), like the atmospheres of Jupiter (90% H_2_ and 10% He), Titan (a moon of Saturn—98% N_2_ and 2% CH_4_), and Mars (approximately 100% CO_2_ at a pressure of <10 mbar), significant differences emerge relative to Earth’s atmosphere.

The selection of Mars, Titan, and Jupiter for this study reflects their distinct atmospheric compositions and relevance in astrobiological research. Mars and Titan are among the few bodies in the solar system with solid surfaces, making them of particular interest for studying potential habitability. Mars offers a CO_2_-rich atmosphere and low pressure, while Titan has a nitrogen-dominated atmosphere with trace hydrocarbons. These unique environments provide valuable test conditions for the adaptability of terrestrial organisms. Jupiter was included as a representative of the gas giant planets, with its hydrogen-helium-dominated atmosphere serving as a model for understanding how photosynthetic organisms might behave in such extreme, anoxic environments.

Microalgae exhibit remarkable adaptability to various extreme environments [2,3,4]. The ability of microalgae to thrive in extreme environmental conditions has been extensively studied [2,3,4,5]. The microalga *Chlorella vulgaris* is notable for its exceptional tolerance to extreme conditions, including high CO_2_ concentrations. This feature is crucial for environmental and astrobiological applications. Studies have shown that *C. vulgaris* can thrive in atmospheres with up to 60% CO_2_ without significant stress, performing photosynthesis and producing oxygen simultaneously [2]. This resilience makes it suitable for carbon capture applications, such as near power plants. High CO_2_ concentrations also enhance *Chlorella’s* photosynthetic activity, boosting biomass and O_2_ production while protecting its photosynthetic mechanism from photoinhibition [2].

Moreover, Wang et al. [3] have shown that *Chlorella* withstand space-like conditions. When exposed to high altitudes using scientific weather balloons, *Chlorella* cells experienced low temperatures, intense UV radiation, and reduced atmospheric pressure. While these harsh conditions caused significant physiological damage—such as DNA damage, reduced cell viability, and substantial mortality—the microalga was able to survive. Transcriptomic analyses revealed adaptive responses, including the expression of 3292 genes associated with heat shock proteins, antioxidant enzymes, DNA repair systems, and ribosomal proteins [3]. Notably, this study focused on survival rather than active growth or photosynthesis at stratospheric altitudes.

Additionally, under drought conditions, *Chlorella* develops protective mechanisms to mitigate stress effects, such as rapid recovery of photosynthetic activity, increased chlorophyll content, and accumulation of osmotic regulators like sucrose and trehalose [4]. Furthermore, *C. vulgaris* has been investigated for its potential role in bioregenerative life support systems (BLSS) due to its ability to produce essential biomolecules such as proteins, lipids, vitamins, and minerals [6].

Zerveas et al. [5] demonstrated that microalga *Scenedesmus obliquus,* in closed systems under anoxic conditions, can alter the atmosphere by enriching it with oxygen (O_2_) through photosynthesis using solar energy. Specifically, when exposed to atmospheres containing 100% nitrogen (N_2_) or 100% helium (He), devoid of CO_2_ and O_2_, the microalga seems to metabolize organic reserves, such as starch, through respiration to produce CO_2_. This CO_2_ is then used in photosynthesis to generate O_2_. Part of the produced O_2_ enriches the atmosphere, while the rest supports metabolic processes like respiration and biomass production. The recycling of products and substrates between photosynthesis and respiration contributes to an oxygen-enriched atmosphere and the microalgae’s survival.

Microalgae are photosynthetic organisms capable of harnessing solar energy and absorbing large amounts of carbon dioxide, thus contributing to the production of atmospheric oxygen on Earth. *Chlorella vulgaris* has served as an essential model organism due to its simple structure, rapid growth under autotrophic, mixotrophic, and heterotrophic conditions, and high photosynthetic efficiency [7]. *C. vulgaris* consists of microscopic spherical cells with diameters ranging from 2 to 10 μm. The reproduction of *C. vulgaris* is asexual and occurs through a process called autosporulation. During autosporulation, the parent cell produces daughter cells within its cell wall before releasing them into the environment [7,8].

The aim of this study is to investigate the resilience and metabolic functionality of the unicellular eukaryotic microalga *C. vulgaris* under conditions simulating the atmospheres of Mars, Jupiter, and Titan. Specifically, it examines whether this microalga can survive, photosynthesize, and grow in extreme extraterrestrial atmospheres significantly different from Earth’s. The findings will deepen our understanding of the microalga’s immediate adaptive mechanisms in extreme conditions, highlighting its astrobiological and astrobiotechnological potential.

## 2. Materials and Methods

### 2.1. The Microalga Chlorella vulgaris—Culture Conditions and Experimental Procedures

A wild type strain of the unicellular green alga *Chlorella vulgaris* (SAG Number 211-11b) (Beyerinck, 1890) was cultured using Bold Basal Medium (BBM) as the nutrient medium [9]. Initial *C. vulgaris* cultures were prepared in 250 mL glass tubes shaped like saxophones (dimensions: 5 cm × 50 cm). The “saxophones” had an open top covered with hydrophobic cotton, and an air pump that provided continuous airflow (~50 L/h) through a tube at the bottom, ensuring optimal aeration and mixing to prevent sedimentation and enhance nutrient exchange (Supplementary Figure S1). These cultures were maintained under constant light intensity (about 50 μmol m^−2^ s^−1^) and room temperature (25 °C) for one week, serving as mother cultures for subsequent experimental procedures.

Both autotrophic and mixotrophic BBM media were prepared for experimental procedures. The autotrophic medium was identical to the original BBM, while the mixotrophic medium included an additional 5 g/L glucose to provide an external organic carbon source. All experiments were conducted in hermetically sealed 125 mL glass vials with septa (dimensions: 5 cm × 9.5 cm), simulating the atmospheric conditions of Earth (atmospheric air—control), Jupiter (90% H_2_ and 10% He), Titan (98% N_2_ and 2% CH_4_), and Mars (about 100% CO_2_). Each vial contained 50 mL of the medium and 75 mL of the gaseous phase. The initial cell concentration was 1 μL PCV (packed cell volume)/mL. In all cases except the Mars simulation, the atmospheric pressure was approximately 1000 mbar. For Mars, the pressure was much lower (<10 mbar), achieved using a SpeedVac Vacuum Concentrator [10]. To establish a 100% CO_2_ atmosphere, a compressed CO_2_ gas cylinder was used. Using two sterilized needles (one for gas inflow and the other for outflow), the bottles were filled while maintaining a continuous gas flow to degas the medium and completely replace the air atmosphere. It took approximately 2 min to completely replace the bottle’s atmosphere with CO_2_. The atmospheric composition inside the bottle was verified using gas chromatography with thermal conductivity detection (GC-TCD). To achieve extremely low pressures nearing a vacuum (<10 mbar), a Heto freeze-dryer system (Heto, Maxi Dry Lyo model, Allerod, Denmark) equipped with a vacuum pump was employed. This system is capable of simulating pressures close to a vacuum (<10 mbar). After establishing the desired atmospheric composition in each closed culture system (100% CO_2_), the septum-sealed bottles were placed inside the SpeedVac chamber. During the initiation of vacuum incubation (<10 mbar), a syringe was inserted into the septum of each bottle. This allowed the pressure of the atmosphere inside the bottles to adjust to match the atmospheric pressure of the SpeedVac chamber (<10 mbar) without altering the composition of the atmosphere within the bottles (100% CO_2_). Under these conditions, the liquid medium began to evaporate slowly and gradually under low pressure. To address this issue, we stopped the vacuum after approximately five days of incubation, replenished the evaporated water with distilled water, repeated the gas phase formation process (100% CO_2_) as described earlier, and resumed incubation until the 12th day.

For Jupiter’s (90% H_2_ and 10% He) and Titan’s (98% N_2_ and 2% CH_4_) atmospheres, cultures were initially prepared using 100% H_2_ and 100% N_2_, respectively. To create Jupiter’s atmosphere, 10% of the 100% H_2_ atmosphere was removed using a gas-tight syringe, and 10% He was added. Similarly, for Titan’s atmosphere, 2% of the 100% N_2_ atmosphere was removed and 2% CH_4_ was introduced. The atmospheric composition inside the bottle was verified using gas chromatography with thermal conductivity detection (GC-TCD).

We note that this study aimed to replicate the gas compositions and pressures of these extraterrestrial environments while maintaining laboratory temperatures (25 °C) conducive to sustaining *C. vulgaris*, as the goal was to assess the resilience of the organism’s photosynthetic and metabolic activity under these atmospheric conditions.

### 2.2. Fluorescence Induction Measurement (JIP-Test)

Fluorescence induction was measured using the Plant Efficiency Analyzer (Handy PEA, Hansatech Instruments, King’s Lynn, Norfolk, UK). The JIP-Test is a widely used technique in plant physiology to evaluate the efficiency of the photosynthetic apparatus, particularly of Photosystem II (PSII).

During photosynthesis, only part of the energy absorbed by pigments is used for photochemical processes, while the rest is emitted as heat or fluorescence. Fluorescence induction in photosynthetic organisms, first observed by Kautsky and Hirsch [11], exhibits two phases: rapid and slow [12]. Today, analyzing the fluorescence induction curve, particularly the rapid phase, is a tool for investigating the structure and function of the photosynthetic apparatus.

Rapid fluorescence phase measurements were recorded at 10 μs intervals over one second. Fluorescence was measured at a 12-bit resolution, with excitation via three red-light LEDs (intensity 3000 μmol m^−2^ s^−1^). For fluorescence induction measurements, we placed the flat bottom of the small culture bottle (Ø 5 cm) directly on the PEA sensor using a specialized adapter that ensured complete darkness for at least 5 min, allowing the PSII reaction centers to fully open before exposure to saturated light. Under these conditions, and without opening the culture bottles, we conducted measurements of the culture in ambient conditions using the JIP-test [13]. Data analysis was conducted using Biolyser HP 4.0 software.

Key parameters studied included the Fv/Fm ratio (indicative of the maximum quantum efficiency of photosystem II and overall photosynthetic efficiency), Sm (a measure of the electron transport reservoir size), dV/dt_0_ (electron transfer speed), N (number of Q_A_ reduction cycles required to close all reaction centers), TR_0_/RC (energy trapped per reaction center), ET_o_/RC (electron flow per reaction center), RC/CS_0_ (density of active reaction centers per cross-sectional area), ABS/RC (functional antenna size per reaction center), DI_0_/RC (non-photochemical energy dissipation per active center), and PI(abs) (a performance index combining the efficiency of energy absorption, trapping, and electron transport). Additionally, it is important to note that chlorophyll a fluorescence, particularly the steady-state fluorescence compared to the transient peak, provides complementary information to O_2_ evolution. This relationship underscores that the fluorescence measurements reflect overall photosynthetic activity, not solely the status of photosystems.

### 2.3. Gas Chromatography—Thermal Conductivity Detection (GC-TCD) for Photosynthetic Activity

For qualitative and quantitative gas analysis, particularly oxygen, in closed culture systems, gas chromatography using thermal conductivity detection (GC-TCD) (Shimadzu GC 2010 Plus, Kyoto, Japan) was employed, with argon as the carrier gas at 5 bar pressure. A 250 μL gas sample was injected using a gas-tight precision syringe. Gases (O_2_ and N_2_) were separated based on thermal conductivity differences. The thermal conductivities of argon, nitrogen, and oxygen were 0.0001772 W/cmK, 0.0002598 W/cmK, and 0.0002674 W/cmK, respectively. A long capillary column (Ø 5 Å) was used, with detector temperature maintained at 200 °C, oven at 120 °C, and injection point at 180 °C. Quantitative gas analysis was performed using reference curves derived from known gas concentrations. According to the GC-TCD measurements for photosynthetic activity estimation, we recorded O_2_ production during the first 2 days of incubation time (GC-TCD measurements at 0 and 2 days). Additionally, we know the biomass in the closed bottles (in μL PCV/mL) and the photosynthetic rate, expressed as mLO_2_·mL PCV^−1^·day^−1^.

### 2.4. Measurement of Packed Cell Volume (PCV)

To measure the packed cell volume of individual cultures, 1000 μL samples were centrifuged in calibrated capillary tubes for 5 min at 1500 g to sediment the cells [10]. Cellular concentration was expressed as packed cell volume (PCV) per mL of culture. This method assessed the growth rate of microalgal cultures under different conditions.

### 2.5. Data Analysis

All the experiments were conducted with a minimum of three repetitions to ensure reliability. Error bars on the diagrams represent the standard error of the mean (SEM), providing a measure of variability and precision in the sample means.

## 3. Results

### 3.1. Resilience of the Autotrophic Culture Chlorella vulgaris in the Atmospheres of Mars, Jupiter and Titan

This experiment examined the ability of the eucaryotic unicellular green microalga *Chlorella vulgaris* to survive and maintain its photosynthetic activity under conditions simulating the atmospheres of Jupiter, Titan (a moon of Saturn), and Mars. To assess whether the microalga could survive in these extraterrestrial environments, several parameters were monitored over time. These included the functional performance of the photosynthetic apparatus through chlorophyll a fluorescence induction measurements (JIP-test), its ability to metabolize and produce oxygen via photosynthesis using gas chromatography with thermal conductivity detection (GC-TCD), and its capacity to multiply assessed by measuring the growth of the cultures, expressed in μL PCV/mL. The Earth’s atmosphere was used as a control, and the experiments were conducted under both autotrophic and mixotrophic conditions. Measurements were taken after incubating the cultures in the different atmospheres for 2, 5, and 12 days.

The atmospheres of the other celestial bodies differ significantly from that of Earth. The compositions used were as follows: Earth’s atmosphere consisted of 78% N_2_, 21% O_2_, and 0.04% CO_2_; Jupiter’s atmosphere contained 90% H_2_ and 10% He; Titan’s atmosphere consisted of 98% N_2_ and 2% CH_4_; and Mars’ atmosphere was composed of approximately 100% CO_2_ at a pressure of <10 mbar. In all experimental setups, fluorescence induction curves were examined to provide fundamental insights into photosynthetic efficiency under autotrophic conditions in different planetary environments.

**Figure 1:**
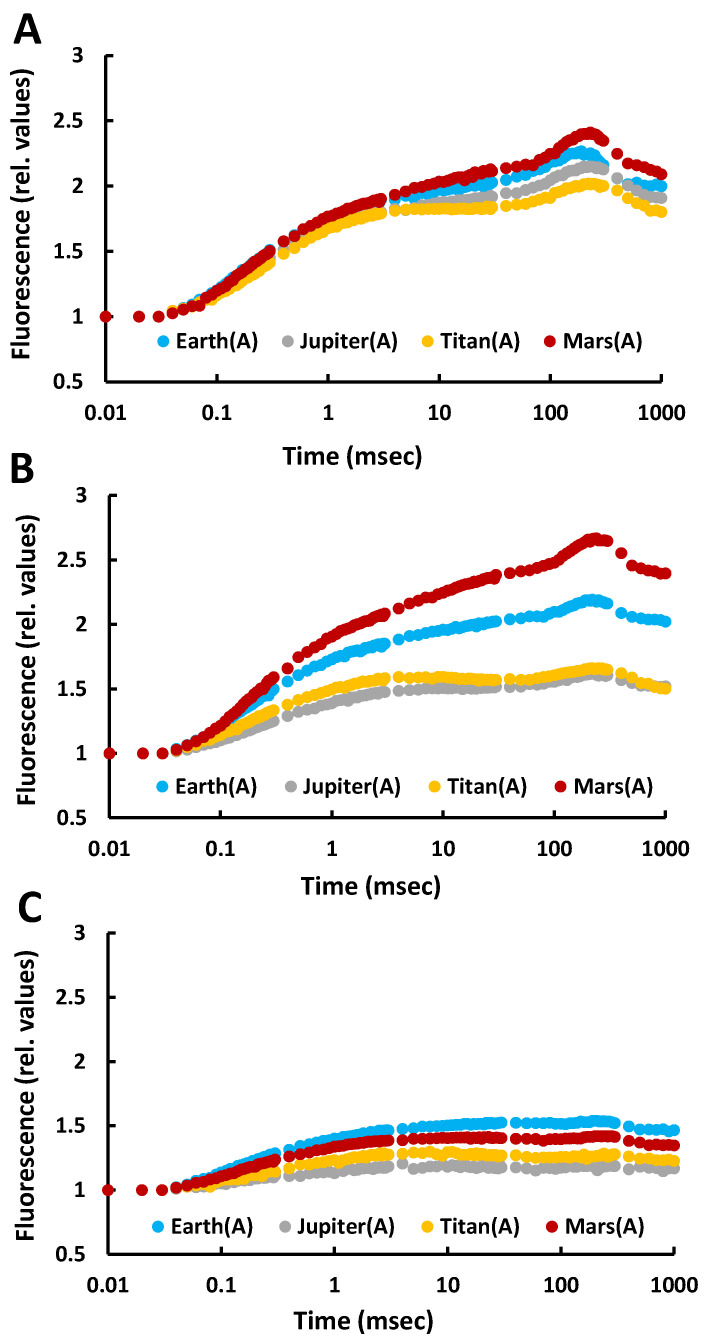
Fluorescence induction curves for all treatments with autotrophic cultures of the microalga *Chlorella vulgaris* under different simulated atmospheres (Jupiter, Titan, Mars) compared to the control treatment (Earth atmosphere) after incubation under these conditions for (**A**) 2 days, (**B**) 5 days, and (**C**) 12 days.

Fluorescence induction measurements reflect the functionality of the photosynthetic apparatus. On day 2, the fluorescence curves were nearly identical across all atmospheres and retained the characteristic shape of the OJIP curve observed in the control (Earth). This indicated proper functioning of the photosynthetic mechanism and efficient electron flow despite the extreme atmospheric conditions, suggesting that *C. vulgaris* under autotrophic conditions was capable of surviving and maintaining photosynthetic activity under these anoxic extreme conditions (Figure 1).

By day 5, the fluorescence curves began to diverge between atmospheres. The Earth’s curve showed a slight decline but retained its characteristic shape, whereas those for Jupiter and Titan displayed noticeable drops, indicating stress on the cells. However, the curves still maintained the characteristic OJIP profile. On Mars, the fluorescence curve exceeded that of Earth, likely due to the CO_2_ rich atmosphere (100%) combined with low atmospheric pressure (<10 mbar), which appeared to enhance photosynthesis (Figure 1).

By day 12, the curves had decreased in amplitude and steepness across all atmospheres, but still indicated photosynthetic activity. The overall decline, including in Earth conditions, was attributed to nutrient depletion in the closed system rather than atmospheric composition. These findings suggest that *C. vulgaris* can survive and photosynthesize in the atmospheres of Jupiter, Titan, and Mars under autotrophic conditions without prior adaptation. However, it is important to note that these results are based on controlled simulations and do not account for the absence of liquid water in such environments.

**Figure 2:**
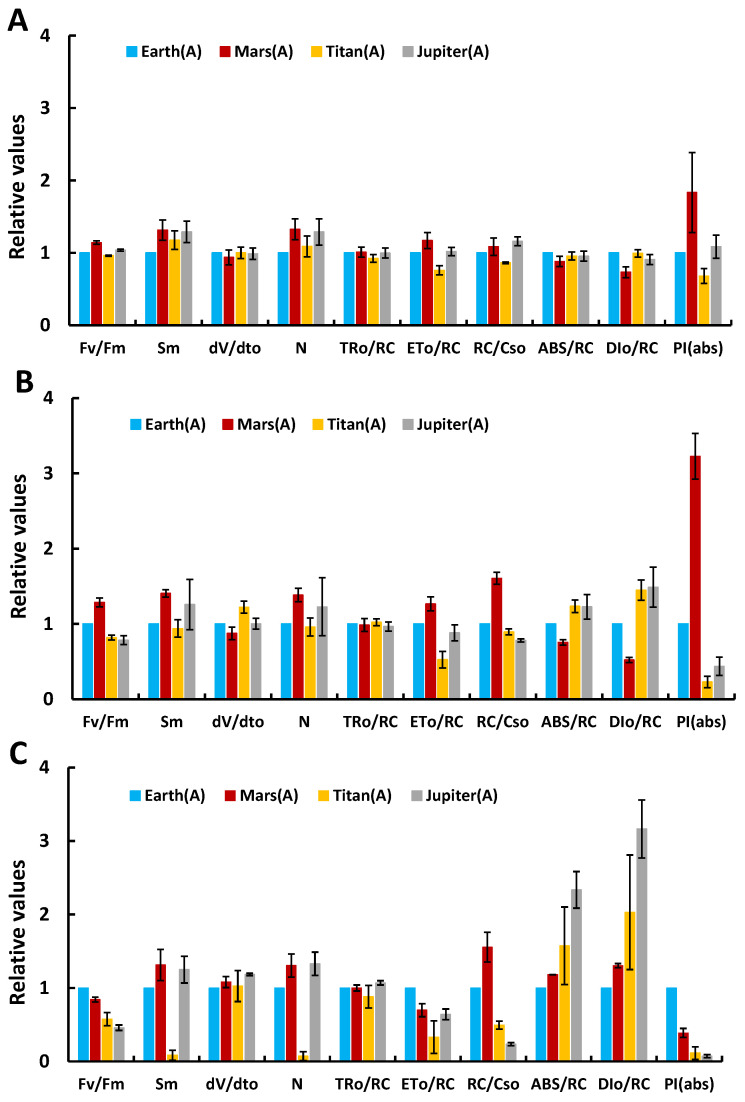
Differentiation of selected parameters from the JIP test (highlighting the structure and function of the photosynthetic mechanism) for treatments under simulated Jupiter, Titan, and Mars atmospheres compared to the control treatment (Earth atmosphere) under autotrophic conditions, after incubation under these conditions for (**A**) 2 days, (**B**) 5 days, and (**C**) 12 days.

Further investigations examined structural and functional changes in the photosynthetic mechanism across conditions using the JIP-test. Figure 2 presents normalized parameter values relative to the control (Earth). Key parameters studied included the Fv/Fm ratio (indicative of photosynthetic efficiency), Sm (a measure of the electron transport reservoir), dV/dt_0_ (electron transfer speed), N (number of Q_A_ reduction cycles), TR_0_/RC (energy trapped per reaction center), ET_o_/RC (electron flow per reaction center), RC/CS_0_ (active reaction center density), ABS/RC (functional antenna size), DI_0_/RC (non-photochemical energy dissipation per active center), and PI(abs) (photosynthetic efficiency).

On day 2, the Fv/Fm ratio on Mars was higher than that on Earth, indicating increased photosynthetic efficiency, likely due to the high CO_2_ concentration [10]. In Titan’s atmosphere, Fv/Fm was slightly lower than Earth’s, while in Jupiter’s atmosphere, it was slightly elevated. Sm values were higher for all three extraterrestrial atmospheres compared to Earth, reflecting a larger electron transport reservoir (the number of available electron carriers between the reaction centers of photosystem II and the plastoquinone pool). The dV/dt_0_ values were similar across all atmospheres, suggesting functional and healthy photosystems. The RC/CS_0_ and ABS/RC values were also comparable to Earth’s, indicating no stress. PI(abs), a sensitive parameter reflecting PSII functionality, showed comparable values between Earth, Jupiter, and Titan, with Mars exhibiting significantly higher values, indicating robust photosynthetic activity even after 48 h of incubation in extreme conditions.

On day 5 (120 h of incubation), parameter values across the planets showed no significant deviations from Earth’s normalized values, except for PI(abs) and DI_0_/RC. Jupiter and Titan cultures demonstrated slight stress in their photosynthetic apparatus. Stress responses included decreased active center density (RC/CS_0_), increased antenna size (ABS/RC), higher non-photochemical energy dissipation (DI_0_/RC), and reduced Fv/Fm and PI(abs). In contrast, the Mars atmosphere showed enhanced photosynthetic performance, expressed as increased Fv/Fm and PI(abs). These changes suggest that the microalgae remained capable of photosynthesis despite moderate structural and functional adjustments to the PSII.

By day 12 (288 h of incubation), significant gradients emerged between Earth’s parameters and those of other planetary atmospheres, reflecting stress without PSII collapse. All cultures, even those in Earth-like conditions, exhibited stress, attributed to nutrient depletion in the closed system. These stressors influenced PSII efficiency, electron transport, and energy management. However, the photosynthetic apparatus remained functional across all conditions.

**Figure 3:**
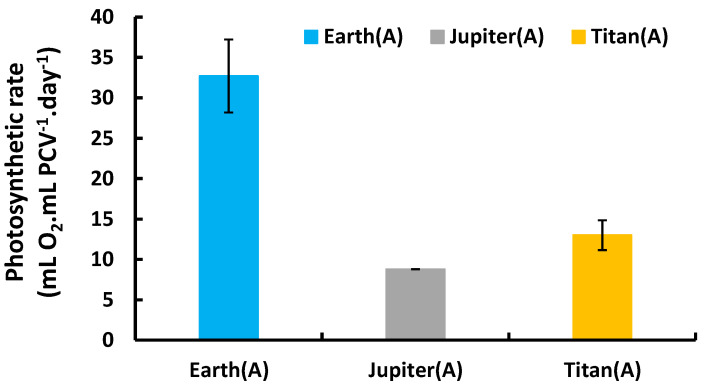
Photosynthetic O_2_ production rate of *Chlorella vulgaris* under autotrophic conditions in different atmospheres.

To confirm metabolic functionality, net photosynthesis was measured (expressed as mL O_2_·mL PCV^−1^·day^−1^) using GC-TCD, except under Mars conditions where low pressure prevented measurement. The results showed oxygen production in all tested atmospheres. Earth’s atmosphere supported the highest photosynthetic activity, followed by Titan and then Jupiter. This confirmed active metabolism and oxygen production by the microalgae despite adverse atmospheric conditions (Figure 3).

**Figure 4:**
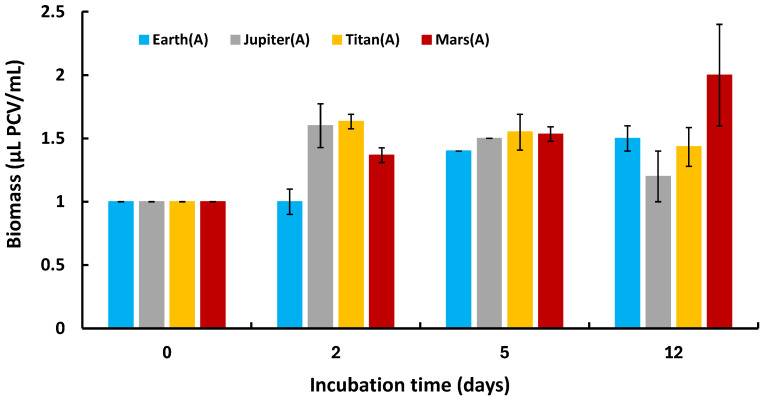
Biomass differentiation kinetics of *Chlorella vulgaris* for all treatments under autotrophic conditions.

Lastly, we assessed the ability of *C. vulgaris* to multiply under these extreme conditions by monitoring biomass changes over time, expressed as μL PCV/mL. All cultures started at 1 μL PCV/mL and exhibited biomass growth over time, indicating active proliferation in all atmospheric conditions (Figure 4). The distinct biomass production trends observed under autotrophic conditions in the reducing atmospheres of Jupiter and Titan compared to the oxidizing conditions of Earth and Mars suggest potential shifts in metabolic pathways. In the methane-rich atmosphere of Titan, Chlorella vulgaris may partially rely on alternative metabolic processes, such as fermentative or anoxygenic pathways, to build biomass, which could explain the surge followed by a plateau. This interpretation is supported by the inverse relationship between biomass production (Figure 4) and oxygen production (Figure 3) under these conditions.

These findings suggest the potential capability of microalgae to survive, photosynthesize, and reproduce under the simulated extreme atmospheres of Jupiter, Titan, and Mars. However, it is important to note that these results are based on controlled simulations and do not account for the absence of liquid water in such environments.

### 3.2. Resilience of Mixotrophic Chlorella vulgaris Cultures in the Atmospheres of Mars, Jupiter and Titan

**Figure 5:**
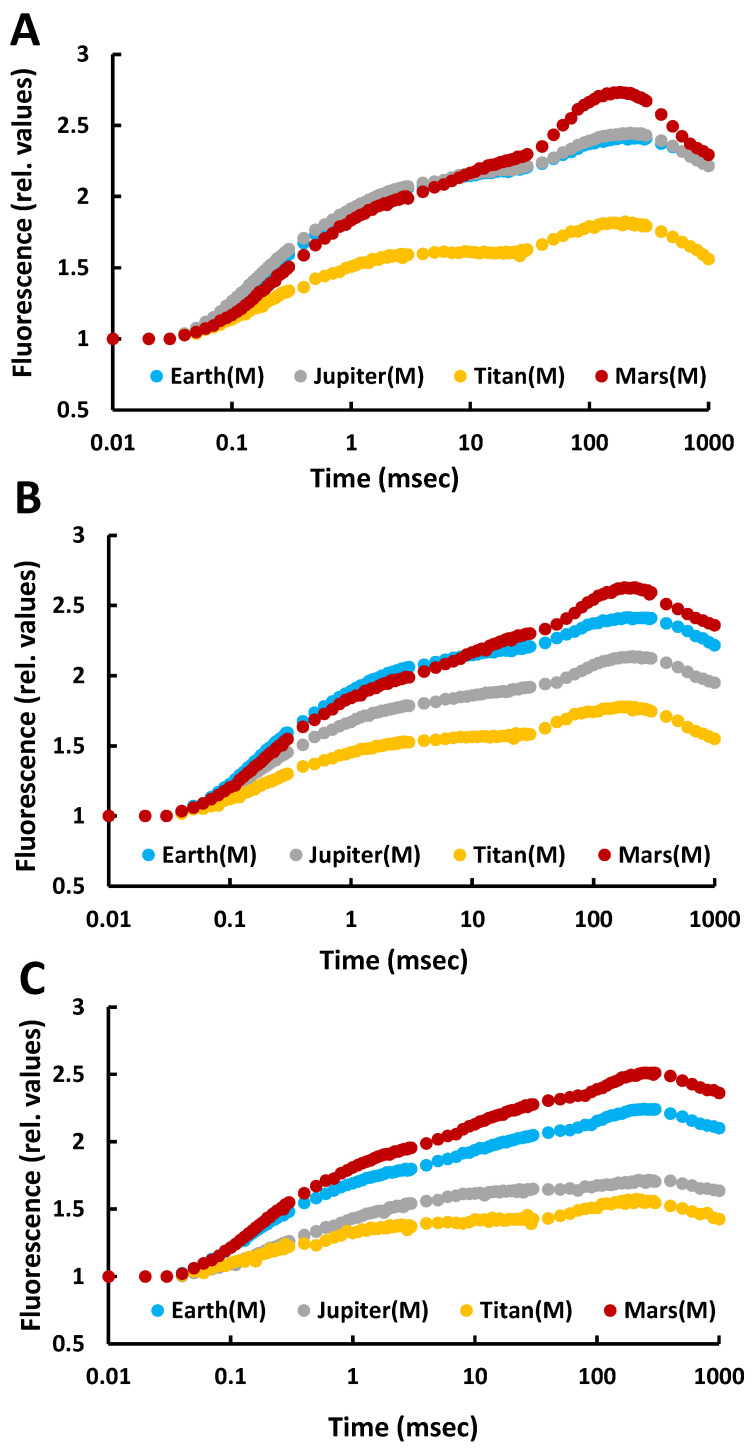
Fluorescence induction curves of chlorophyll for all treatments with mixotrophic cultures of the microalga *Chlorella vulgaris* under different simulated atmospheres (Jupiter, Titan, Mars) compared to the control treatment (Earth atmosphere), after incubation under these conditions for (**A**) 2 days, (**B**) 5 days, and (**C**) 12 days.

We conducted experiments under mixotrophic conditions by supplementing inorganic nutrients with glucose (5g/L) to examine the behavior of mixotrophic cultures in various planetary atmospheres. The fluorescence induction curves of *C. vulgaris* under mixotrophic conditions in four different atmospheres (Earth, Jupiter, Titan, Mars) and after different incubation periods (2, 5, and 12 days) were comparable to or even higher than those observed under autotrophic conditions (Figure 5). This indicates that the microalga retains a highly active photosynthetic system. The fluorescence induction curve under Earth’s atmosphere was sharp and high, nearly overlapping with the curve observed under Jupiter’s atmosphere. The Mars curve was the highest among all atmospheres, while Titan’s curve was the lowest. These findings suggest a healthy and active photosynthetic mechanism in the microalga (Figure 5A).

By Day 5, the fluorescence curves remained almost unchanged from Day 2. The Mars atmosphere continued to exhibit the highest fluorescence curve, while the curves for Earth and Titan remained at similar levels, and Jupiter’s curve showed a slight decline (Figure 5B). By Day 12, the fluorescence curves under mixotrophic conditions were similar to those observed on Day 5, albeit slightly reduced (Figure 5C). These results suggest that the microalgae can maintain its photosynthetic capacity over longer periods when utilizing both light and an external organic carbon source, regardless of the incubation atmosphere.

In addition to the fluorescence induction curves, we evaluated JIP-test parameters related to the structure and functionality of the photosynthetic mechanism under mixotrophic conditions. This was performed to better determine the functionality of the photosynthetic system in extraterrestrial atmospheres. The values of the parameters were normalized to the control (Earth). The same parameters evaluated under autotrophic conditions were examined, including Fv/Fm, Sm, dV/dt_0_, N, TR_0_/RC, ET_0_/RC, RC/CS_0_, ABS/RC, DI_0_/RC, and PI(abs). These parameters provided a comprehensive view of the photosynthetic activity of *C. vulgaris* under mixotrophic conditions in different planetary atmospheres.

**Figure 6:**
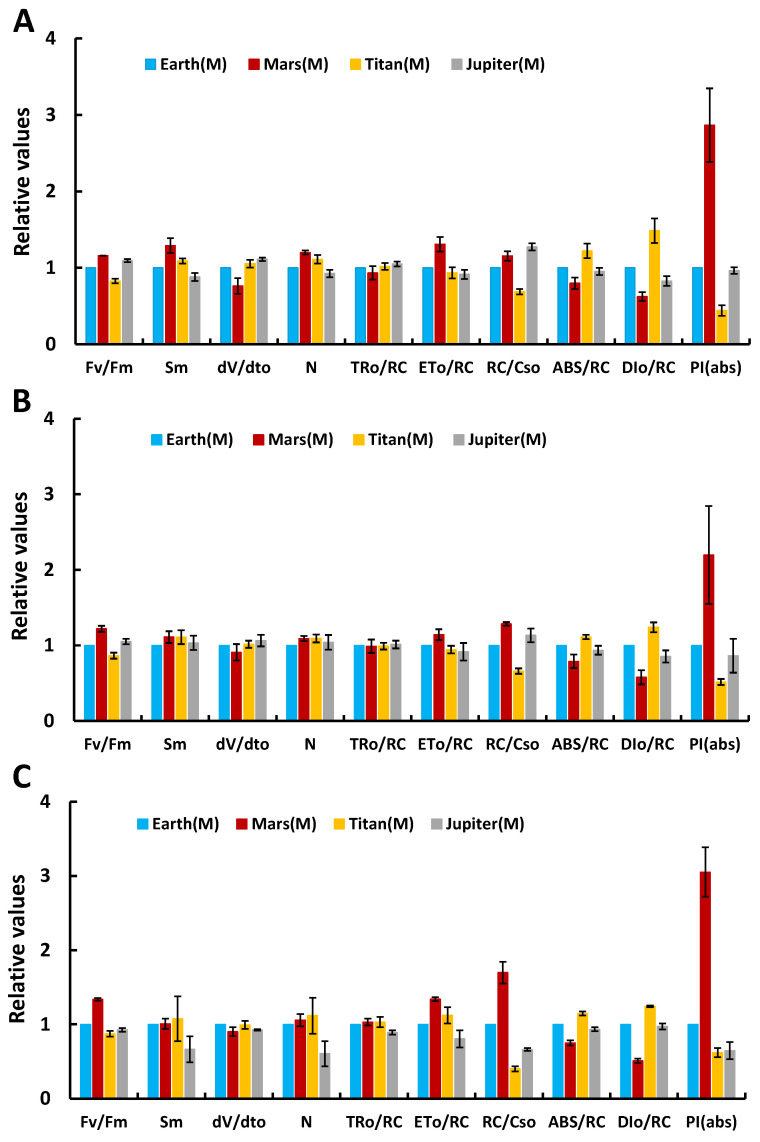
Differentiation of selected parameters from the JIP test (highlighting the structure and function of the photosynthetic mechanism) for treatments under simulated Jupiter, Titan, and Mars atmospheres compared to the control treatment (Earth atmosphere) under mixotrophic conditions, after incubation under these conditions for (**A**) 2 days, (**B**) 5 days, and (**C**) 12 days.

On Day 2, all parameters were nearly identical to the normalized Earth values (Figure 6A). Several Mars parameters were higher than those of Earth, with the PI(abs) parameter being significantly higher for Mars compared to Earth, while the DI_0_/RC ratio was considerably lower. This indicates that the photosynthetic efficiency of the microalga in a Martian atmosphere is high, with low non-photochemical energy dissipation.

On Day 5, the parameters remained nearly unchanged compared to Earth and Day 2 values. The Sm and dV/dt_0_ parameters for the other planets were similar to the control (Earth), indicating a large electron transfer pool and high electron transfer rate. This demonstrates that the photosynthetic mechanism continues to function efficiently (Figure 6B). By Day 12, the deviations among the parameters of the various planets were slightly more pronounced. However, the recorded stress was minimal and significantly less than that observed under autotrophic conditions (Figure 6C).

**Figure 7:**
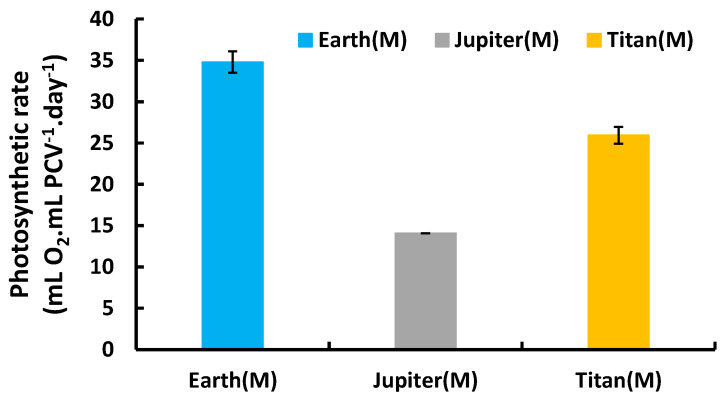
Net photosynthesis rate of *Chlorella vulgaris* under mixotrophic conditions in different atmospheres.

To confirm the functionality of the microalga’s metabolism under mixotrophic conditions, we measured photosynthetic activity (net photosynthesis), expressed as mL O_2_·mL PCV^−1^·day^−1^, using gas chromatography with a thermal conductivity detector (GC-TCD). The results showed significant photosynthetic activity in all extraterrestrial atmosphere experiments under mixotrophic conditions (except for Mars, where the extremely low atmospheric pressure made GC-TCD measurements impossible). The highest O_2_ production rate was observed in Earth’s atmosphere, followed by Titan and then Jupiter (Figure 7). Additionally, photosynthetic rates under all extraterrestrial conditions were higher than those observed under autotrophic conditions (Figure 7).

**Figure 8:**
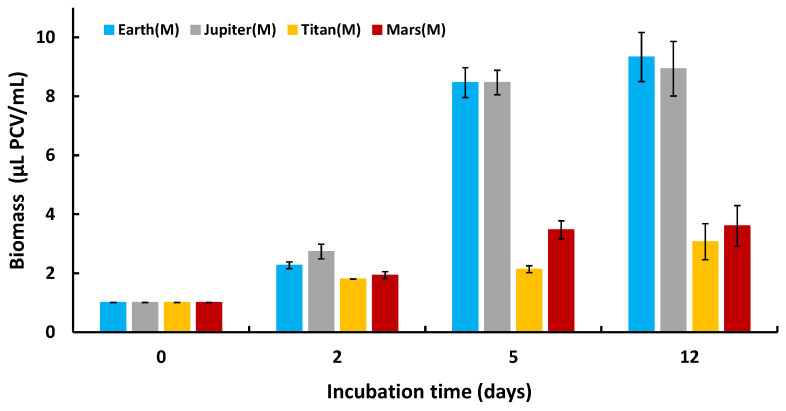
Biomass differentiation kinetics of *Chlorella vulgaris* for all treatments under mixotrophic conditions.

Tracking biomass differentiation over time revealed that *C. vulgaris* cultures grown under mixotrophic conditions in various planetary environments for 2, 5, and 12 days exhibited greater growth than under corresponding autotrophic conditions. This confirms the metabolic functionality of the microalga in all experiments (Figure 8). All measurements on Day 0 began with 1 μL PCV/mL. Over subsequent days, the biomass increased significantly compared to cells grown in autotrophic nutrient media. This is due to the greater energy production enabled by the presence of organic carbon in the medium, leading to higher cell division rates and biomass differentiation between autotrophic and mixotrophic cultures. Biomass growth was observed in all planetary environments, not just Earth, demonstrating that the cells can not only survive but also proliferate in extraterrestrial environments under mixotrophic conditions.

## 4. Discussion

This study investigated the ability of the unicellular eukaryotic green alga *Chlorella vulgaris* to survive and metabolize by maintaining its photosynthetic processes under various extraterrestrial atmospheric conditions. These included simulated atmospheres resembling those of Mars, Jupiter, and Titan. Using Earth as a control and comparing autotrophic and mixotrophic conditions across different planetary atmospheres, the experimental approach provided valuable insights into the adaptability (without any acclimation period) and metabolic functionality of this microalga in environments vastly different from Earth.

Analysis of the structure and functionality of the photosynthetic machinery of *C. vulgaris* using the JIP-test revealed that the microalga maintained its photosynthetic efficiency under all simulated atmospheres, including those of Mars, Jupiter, and Titan. Fluorescence induction curves indicated that the microalga’s photosynthetic efficiency remained largely intact (or only marginally stressed) in all atmospheres. Fluorescence induction curves revealed a substantial decline in fluorescence by day 12 across all autotrophic cultures regardless of atmospheric composition, indicating that while the photosynthetic apparatus remained functional, it was significantly stressed. This decline appears to be a generic effect of the closed culture system rather than a direct result of the atmospheric conditions. In contrast, the mixotrophic cultures exhibited more stable fluorescence patterns, likely due to the supplemental energy and carbon provided by glucose, which mitigated these stresses and sustained growth. The similarity between these curves and those of the Earth-like control suggests that the microalga can survive and operate its photosynthetic machinery, without any acclimation period, in entirely foreign, anoxic planetary environments. Specifically, Fv/Fm values, which represent the maximum photosynthetic efficiency of Photosystem II (PSII), showed no significant photoinhibition or PSII stress in any tested environment.

Particularly intriguing were the results from the simulated Martian atmosphere, where the fluorescence curves and Fv/Fm values surpassed those observed under Earth-like conditions, reflecting the health and high photosynthetic performance of the microalga. These findings are further supported by the higher biomass growth observed in purely photosynthesizing cultures under the Martian atmosphere. The elevated Fv/Fm values and fluorescence curves not only highlight the robust photosynthetic performance of *C. vulgaris,* but also align with its enhanced capacity to efficiently utilize the CO_2_-rich, low-pressure environment of Mars for growth. Previous studies have shown that elevated CO_2_ concentrations stimulate photosynthesis in microalgae by enhancing carbon fixation rates. Specifically, *C. vulgaris* exhibits peak photosynthetic activity at 30–40% CO_2_, while concentrations above 60% inhibit photosynthetic efficiency [2]. However, in conditions approximating 100% CO_2_—such as those of the Martian atmosphere—coupled with near-vacuum atmospheric pressure (<10 mbar), the photosynthetic mechanism remained almost unaffected. These findings are consistent with recent studies [10], which suggest that the reduction in atmospheric pressure on Mars may increase thylakoid membrane fluidity, enhancing plastoquinone (PQ) mobility and potentially improving electron transport and photosynthetic activity. However, we also acknowledge that the observed improvement in CO_2_ fixation under Martian conditions may be more simply explained by the elevated CO_2_ concentrations (even at very low atmospheric pressures), which could directly enhance photosynthetic performance independent of pressure effects.

After extended incubation periods of 5 and 12 days in closed systems, deviations in fluorescence curves became more pronounced, particularly in the atmospheres of Jupiter and Titan, indicating signs of stress. This stress cannot be attributed to a lack of O_2_, as these cultures generated their own oxygen through photosynthesis (Figure 3). Instead, it is likely due to imbalances in metabolic processes under these reducing atmospheres. For instance, CO_2_ may be generated via fermentative metabolism, providing a substrate for photosynthesis. In the absence of atmospheric O_2_ or CO_2_, organic reserves within the microalgae can be metabolized through fermentative pathways, producing CO_2_, which is then reinvested in photosynthesis to generate organic matter and O_2_. Previous studies [5] suggest that such conditions hinder or reduce photosynthetic efficiency without completely suppressing it. The initial metabolism of these organic reserves, in the absence of atmospheric O_2_ and CO_2_, appears to rely on nitrogen oxides likely derived from nitrate in the culture medium or other cellular mechanisms, facilitating the production of O_2_ through photosynthesis [14,15]. However, a significant portion of the photosynthetically produced O_2_ is reinvested in respiration, reducing its release into the atmosphere. This explains the lower photosynthetic activity (expressed as mL O_2_·mL PCV^−1^·day^−1^) observed under these extraterrestrial atmospheres compared to Earth-like controls, despite the retention of high photosynthetic efficiency (expressed as Fv/Fm). Such metabolic dynamics also contribute to the more limited growth observed under these conditions.

By day 12, fluorescence curves across all autotrophic conditions (including the control) showed increased stress, likely due to the constraints of closed systems. After 12 days of incubation, nutrients began to deplete as they were consumed by the microalgae. Nutrient limitations can reduce electron transport rates, chlorophyll content, and carbon assimilation, explaining the overall decline in photosynthetic efficiency across all atmospheres, including Earth [16]. Despite this stress, *C. vulgaris* continued to survive and photosynthesize, highlighting the resilience of its photosynthetic machinery in extreme environments.

Under mixotrophic conditions, *C. vulgaris* exhibited enhanced photosynthetic activity across all examined planetary atmospheres compared to autotrophic conditions. The presence of an external organic carbon source such as glucose allowed the cells to utilize both photosynthesis and respiration through feedback between these metabolic pathways. This resulted in increased energy production (compared to autotrophic conditions) and, consequently, higher growth rates [17]. Fluorescence curves consistently showed high photosynthetic activity throughout the experiment (up to the 12th day of incubation). Mixotrophic conditions enable *C. vulgaris* to thrive by utilizing both light and organic substrates for energy production, making it particularly suitable for environments where light may be limited or intermittent [18]. Furthermore, the stability of several JIP-test parameters, such as Sm and dV/dt_0_, under different planetary atmospheres suggests that the electron transport system remained even more efficient under mixotrophic conditions, resulting in enhanced photosynthetic activity and sustained growth.

Our findings build upon previous investigations into terrestrial life’s adaptability to extraterrestrial conditions, which have demonstrated the resilience of methanogenic bacteria [19] and non-methanogenic organisms [20,21] to various atmospheric compositions. Unlike anaerobic systems or those reliant on chemical energy, the photosynthetic activity of *C. vulgaris* highlights the potential of oxygenic photosynthesis to sustain life and recycle atmospheric gases in extraterrestrial habitats. This resilience and adaptability of *C. vulgaris* in extreme (by Earth standards) planetary atmospheres highlights its potential use in bio-regenerative life support systems (BLSS). These systems are designed to regenerate oxygen, water, and food while recycling waste to sustain closed-loop ecosystems, making them crucial for long-term human survival in deep space [22,23]. Cultivation of this microalga could contribute to sustained oxygen production in future astronaut colonies on other planets by consuming the CO_2_ exhaled by astronauts [2,24]. Additionally, the organism is nutrient-rich and considered a superfood [25]. The rapid biomass production, particularly in mixotrophic cultures where external carbon sources are available, underscores the potential for enhancing microalgal productivity in space habitats where light alone may be insufficient for sustained growth.

This study highlights the resilience of *C. vulgaris* in simulated extraterrestrial atmospheric compositions under laboratory conditions, emphasizing its potential for bioregenerative applications in space exploration. While the initial gas compositions and pressures reflected those of Mars, Jupiter, and Titan, the incubation temperature (25 °C) was maintained to support active photosynthesis and metabolism, differing from the actual environmental temperatures of these celestial bodies. Pressure dynamics within the sealed vials likely shifted during the experiments, altering initial conditions. Unlike studies focusing on survival under extreme low pressures, such as methanogens at 6–143 mbar [26], our research examines active metabolic and photosynthetic responses. These findings complement existing research by expanding our understanding of life’s adaptability to extraterrestrial environments.

The findings of this study underscore the versatility of *C. vulgaris*, a common unicellular eukaryotic organism, not only to survive, but also to immediately adapt its photosynthetic mechanism to extraterrestrial environments. The orchestrated ability of this microalga to function in extreme planetary atmospheres, such as those of Mars, Jupiter, and Titan, demonstrates that the microalga can respond with different “metabolic compositions” depending on its environment. These data offer a new perspective on supporting life in space and open new pathways for revisiting the potential transfer of life through the theory of panspermia. This study illustrates that, in the vast universe, even though conditions may appear harsh and the survival of terrestrial microorganisms unlikely, life, as exemplified by *C. vulgaris*, has ways of orchestrating its metabolism to persist in extreme atmospheric conditions [27]. The survival and growth of *C. vulgaris* in non-terrestrial atmospheric conditions redefine the concept of habitable zones and environments, demonstrating that Earth-like life can adapt to a broader range of atmospheric and environmental conditions than previously thought, expanding our criteria for assessing the habitability of exoplanets. However, it is important to note that these results are based on controlled simulations and do not account for the absence of liquid water in such environments.

The discovery that the eukaryotic unicellular green alga *C. vulgaris* can survive and function effectively in the atmospheres of Mars, Jupiter, and Titan holds profound implications for astrobiology, space exploration, and biotechnological applications under extreme conditions.

## 5. Conclusions

The results of this study demonstrate the remarkable resilience of the microalga *Chlorella vulgaris* in extreme extraterrestrial environments, showcasing its ability to survive, photosynthesize, and proliferate under simulated atmospheric conditions of Mars, Jupiter, and Titan. This resilience, observed under both autotrophic and mixotrophic conditions, underscores the potential of *C. vulgaris* as a key biological component for sustaining life in space. Its capacity to maintain photosynthetic activity and growth in atmospheres vastly different from Earth’s highlights its suitability for bioregenerative life support systems, resource utilization, and potential terraforming efforts. Furthermore, the findings contribute valuable insights into the resilience of terrestrial life forms, challenging traditional definitions of habitability and providing a foundation for exploring astrobiological possibilities. By leveraging the unique capabilities of *C. vulgaris*, humanity can take significant strides toward sustainable space exploration, resource-independent missions, and the long-term goal of extraterrestrial colonization.

## Data Availability

The original contributions presented in this study are included in the article material. Further inquiries can be directed to the corresponding author.

## Supplementary Materials

The following supporting information can be downloaded at: https://www.mdpi.com/article/10.3390/life15010117/s1, Supplementary Figure S1: Preparation of *Chlorella vulgaris* cultures for experimental setup.
