# Supplementary material for: Resilience of Chlorella vulgaris to Simulated Atmospheric Gas Compositions of Mars, Jupiter, and Titan
Source: Life (Basel). 2025 Jan 17;15(1):117. doi: 10.3390/life15010117 (PMC11766941; doi:10.3390/life15010117)
Supplement: Supplementary file 1 [file life-15-00117-s001.zip › life-3400574-supplementary.pdf]

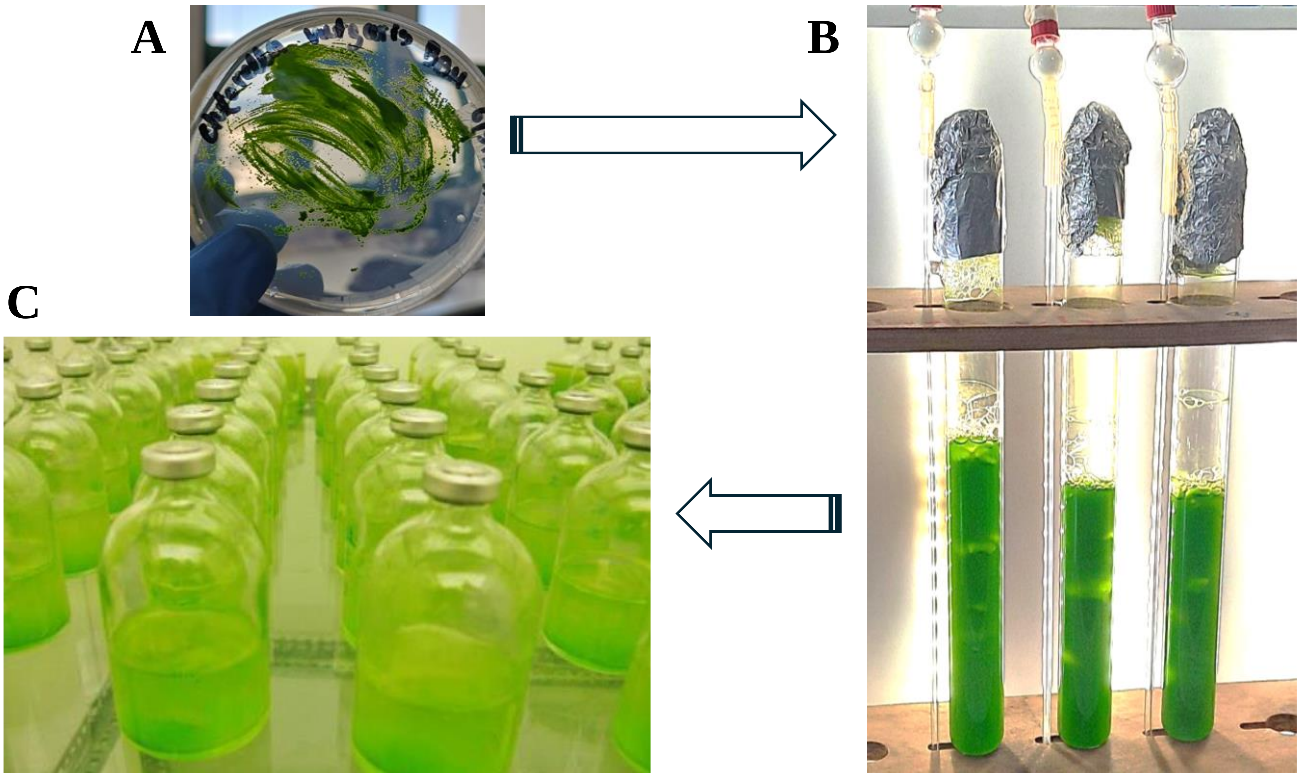

Suppl. Figure S1. Preparation of *Chlorella vulgaris* cultures for experimental setup: *Chlorella vulgaris* from petri plates **(A)** was used to inoculate the initial *C. vulgaris* cultures, which were prepared in 250 mL glass tubes shaped like saxophones (dimensions: 5 cm × 50 cm). The "saxophone" tubes had an open top covered with hydrophobic cotton, and an air pump provided continuous airflow (~50 L/h) through a tube at the bottom, ensuring optimal aeration and mixing to prevent sedimentation and enhance nutrient exchange **(B)**. These cultures were maintained under constant light intensity (approximately 50 μmol m^{-2} s^{-1}) and room temperature (25°C) for one week, serving as mother cultures for subsequent experimental procedures. All experiments were conducted in hermetically sealed 125 mL glass vials with septa (dimensions: 5 cm × 9.5 cm) **(C)**.
